# Health-Related quality of life by 31-item Cervantes scale in breast cancer survivors undergoing adjuvant endocrine therapy

**DOI:** 10.1016/j.clinsp.2024.100324

**Published:** 2024-02-06

**Authors:** Isis Danyelle Dias Custódio, Fernanda Silva Mazzutti Nunes, Mariana Tavares Miranda Lima, Kamila Pires de Carvalho, Andressa Miranda Machado, Paula Philbert Lajolo, Carlos Eduardo Paiva, Yara Cristina de Paiva Maia

**Affiliations:** a Molecular Biology and Nutrition Research Group, School of Medicine, Federal University of Uberlândia, Uberlândia, MG, Brazil; b Department of Clinical Oncology, Clinical Hospital, Federal University of Uberlândia, Uberlândia, MG, Brazil; c Department of Clinical Oncology, Barretos Cancer Hospital, Barretos, SP, Brazil; d Nutrition Course, Graduate Program in Health Sciences, School of Medicine, Federal University of Uberlândia, Uberlândia, MG, Brazil

**Keywords:** Health-related quality of life, Menopause, Cancer survivors, Breast neoplasms, Aromatase inhibitors

## Abstract

**Introduction:**

Instruments to manage adverse effects of endocrine therapy with Aromatase inhibitors (AI) may improve adherence and persistence to treatment and Health-Related Quality of Life (HRQL). The 31-item Cervantes Scale (CS-31) is an HRQL questionnaire with particularities of the perimenopausal and postmenopausal period that could be an appropriate instrument to assess HRQL in Breast Cancer (BC) survivors.

**Objective:**

This study aimed to perform additional validation of the CS-31 for BC survivors undergoing adjuvant endocrine therapy.

**Methods:**

This prospective study was performed at three time points named T0, T1, and T2: initial, intermediate, and final follow-up period, respectively, totaling 24 months of follow-up. At each time point, the participants completed the CS-31, Functional Assessment of Chronic Illness Therapy - Fatigue (FACIT-F), and Hospital Anxiety and Depression Scale (HADS). The internal consistency, construct validity, responsiveness analyses, and known-group validity of CS-31 were evaluated.

**Results:**

This study included 89 postmenopausal women diagnosed with hormone receptor-positive early BC in adjuvant endocrine therapy with AI. The internal consistency was good (Cronbach's alpha = 0.89). Construct validity received a positive rating, with 100% of results consistent with prior hypotheses. A prospective improvement in HRQL was identified for the CS-31 Global Score and FACIT-F Total Score and for most of their domains. Furthermore, women with anxiety and depression by HADS presented worse HRQL by CS-31.

**Conclusion:**

The authors identified that the CS-31 seems to be appropriate for use in oncology medical routine and may help to monitor adverse effects and HRQL of BC survivors during adjuvant endocrine therapy.

## HIGHLIGHTS

• CS-31 seems to be appropriate for use in oncology and may help to monitoring adverse effects and HRQL.

## Introduction

Breast Cancer (BC) is a heterogeneous disease comprised of several subtypes with Hormone Receptor-positive (HR+) BC representing 80% of those diagnosed after menopause [1].

Currently, the adjuvant treatment of postmenopausal early-stage HR+ BC with an Aromatase Inhibitor (AI) is considered the standard care [2], being an important ally in increasing disease-free survival [3]. However, it often causes adverse effects related to the central nervous system such as fatigue, depression, and vasomotor symptoms (hot flashes and night sweats); musculoskeletal symptoms such as arthralgia and osteoporosis; cardiovascular such as hypercholesterolemia and angina; and vulvovaginal symptoms including dryness and dyspareunia [4]. Some of these symptoms are frequent in old age, such as depression [5], or during menopause, particularly vasomotor symptoms [6]. Specifically, in relation to AI, the toxicity can be explained, at least in part, to estrogen depletion [7], a hormone that participates in multiple systems and, therefore, has a potential for generalized toxicity in its deprivation [4].

Adverse effects, arising from or exacerbated by AI use, are negatively associated with adherence and persistence to treatment [8] and health-related quality of life (HRQL) [4]. In this sense, the 31-item Cervantes Scale (CS-31) is a HRQL questionnaire that considers particularities of the perimenopausal and postmenopausal women [9] and may be an appropriate option to assess HRQL in BC survivors during AI use.

The CS-31 is a measurement of patients’ perception of their own health status or HRQL, i.e., an instrument capable of measuring patient-reported outcomes (PRO) [10]. PRO instrument has a higher potential to identify adverse effects of therapy (toxicity monitoring), targets for intervention (symptom control), and allows health professionals to understand how treatment effectiveness can be affected by patient perceptions of toxicity (adherence to treatment) [11].

Thus, the aim of this study was to perform additional validation of the CS-31 for BC survivors during adjuvant endocrine therapy.

## Methods

### Ethics statement, Study design, selection of participants and eligibility criteria

This prospective study was approved by the Human Research Ethics Committee of the Federal University of Uberlandia (n° 1.331.949/15, addendum n° 2.905.835/18) and complies with the Declaration of Helsinki. All participants signed a free and informed consent. This study follows the STROBE Statement.

The study was carried out from January 2016 to August 2018 with postmenopausal BC survivors undergoing adjuvant endocrine therapy with AI at the Clinical Hospital of the Federal University of Uberlandia, Minas Gerais, Brazil. The follow-up time was 24 months, and the face-to-face assessments were performed at three-time points: T0, initial follow-up period; T1, intermediate follow-up period, 12 months after T0; and T2, final follow-up period, 24 months after T0, with interviews carried out by properly trained researchers.

Clinical and sociodemographic data were obtained through the analysis of medical records or interview.

### Sample Size

The sample size of a group of individuals and three measurements was calculated with the G*Power software, version 3.1 (Düsseldorf, Germany) [12]. An F test was conducted using ANOVA repeated measures, based on an effect size f of 0.25, an alpha level of 0.05, and at 80% power, a total of 28 women required at each time.

This study included postmenopausal breast cancer survivors undergoing adjuvant endocrine therapy with Aromatase Inhibitors (AI). Participants were recruited at any stage of AI treatment through non-probabilistic convenience sampling. Volunteers were consecutively recruited to minimize selection bias.

**Figure 1:**
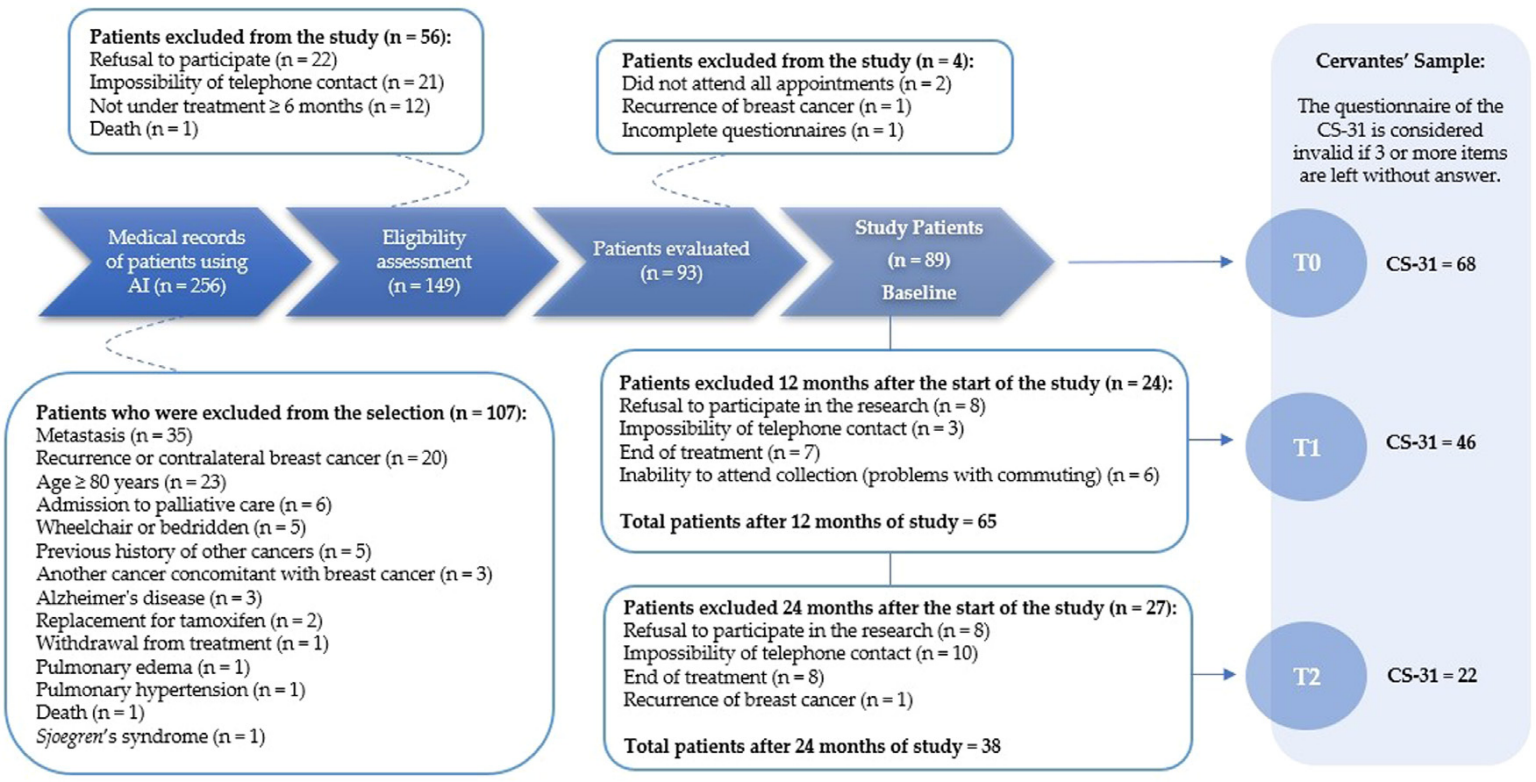
Diagram reporting the number of survivors recruited and selected in the study, and the Cervantes’ sample. Time point: T0, Initial follow-up period; T1, Intermediate period, corresponding to 12-months after T0; and T2, Final follow-up period, corresponding to 24-months after T0; CS-31, 31-item Cervantes Scale.

Initially, 256 patients were selected to participate. After analyzing medical records, 107 patients were excluded, resulting in a final sample size of n = 149. Following the eligibility assessment, 56 patients were further excluded, leaving 93 patients for evaluation. Subsequently, four patients were excluded due to recurrence of breast cancer, incomplete questionnaires, or failure to attend all appointments. In total, 89 patients formed the baseline for the study. Detailed information on survivor recruitment and selection, along with Cervantes' sample recruitment, is provided in Figure 1.

### 31-item Cervantes Scale

**Figure 2:**
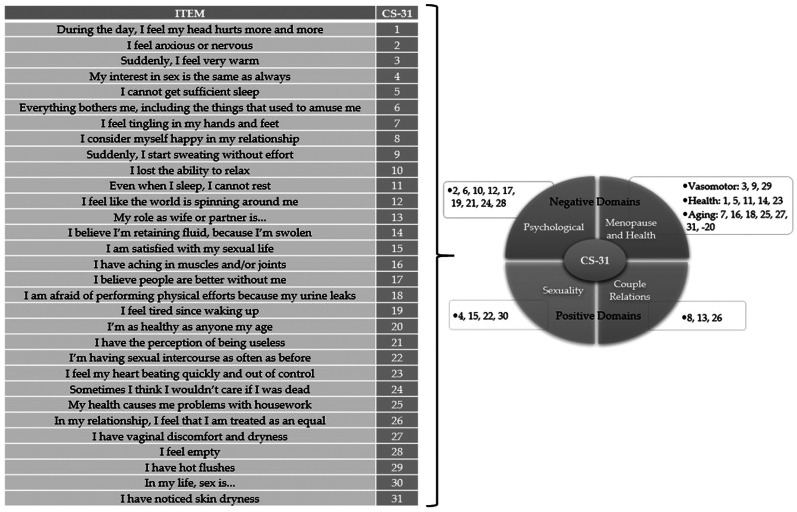
Items contained in the 31-item Cervantes Scale (CS-31).

All participants replied by interview to the CS-31. Figure 2 provides the items contained in this instrument.

The CS-31 was developed in 2004 [9] and a Brazilian Portuguese version validated in 2012 [13], with Cronbach's alpha for the global score of 0.91 and 0.83, respectively. This instrument has been validated in a non-cancer population, but the authors would like to see if it would be useful in a population of BC survivors undergoing adjuvant endocrine therapy.

The CS-31 consists of 31 items rated on a Likert scale from 0 to 5 and divided into four domains, namely Menopause and Health (subdivided into Vasomotor Symptoms, Health, and Aging), Sexuality, Couple Relations, and Psychological. The global score can range from 0 to 155 points, with a higher score representing a worse HRQL. As recommended, the questionnaires were considered invalid if three or more questions were left unanswered. But, if one or two unanswered questions, the score was obtained by multiplying by a correction factor [9].

### Psychometric evaluation

All participants replied by interview to the Functional Assessment of Chronic Illness Therapy - Fatigue (FACIT-F) and Hospital Anxiety and Depression Scale (HADS), which are validated instruments and already well-established for BC patients.

### Validation instruments

#### FACIT-F (version 4)

This scale of 40 items includes the 27-item Functional Assessment of Cancer Therapy-General (FACT-G) that assesses the HRQL and 13 items that assess self-reported fatigue [14]. This instrument measures four well-being subscales (physical, social/family, emotional, and functional), and one fatigue subscale, and derives to calculate the FACIT-F Trial Outcome Index (TOI) (score range 0‒108), the FACT-G total score (score range 0‒108) and the FACIT-F total score (score range 0‒160). Items are rated on a Likert scale from 0 (not at all) to 4 (very much). A higher score represents a better HRQL. The FACIT-F has been previously validated in Brazil [15]. In the present study, the Cronbach's alpha was FACIT-F α = 0.93 (95% Confidence Interval [95% CI] = 0.90–0.95), FACIT-F TOI α = 0.93 (95% CI = 0.90–0.95) and FACT-G α = 0.87 (95% CI = 0.82–0.90).

#### HADS

This self-reported questionnaire comprised seven items targeting anxiety (subscale HADS-A) and seven items targeting depression (subscale HADS-D) [16]. Items are rated using a 4-point Likert-type scale with scores of 0 (minimally present) to 3 (maximally present). The scores range for HADS-A and HADS-D from 0 to 21, with higher scores indicating greater distress. The authors adopted the following cut-off for both scales: < 8 for non-cases, ≥ 8 for doubtful cases, and ≥ 11 for the identification of cases [16]. The HADS has been previously validated in Brazil [17]. In this study, Cronbach's alpha was HADS-A α = 0.76 (95% CI = 0.67–0.83) and HADS-D α = 0.80 (95% CI = 0.72–0.85).

#### Statistical analysis

Internal consistency was studied considering the global score, including all items, and for each domain. The authors use Cronbach's alpha coefficient, considering adequate values between 0.70 and 0.95 [18].

Convergent analyses were assessed by determining the correlation between the CS-31 and a specifically related measure from FACIT-F (global score and domains). The authors hypothesized that the global score between CS-31 and FACIT-F, as they are general measures, as well as constructs indirectly related (Sexuality and Couple Relations of CS-31 with Social/Family Well-Being of FACIT-F) should correlate with *r* > 0.4. The present hypothesis was correlations with *r* > 0.6 due to similar constructs, such as the Psychological of CS-31 and the Emotional Well-Being of FACIT-F. Construct validity is given a positive rating if at least 75% of the results are consistent with prior hypotheses [18]. The authors defined the strength of the correlation coefficient as weak if *r* < 0.4; moderate if *r* is ≥0.4 and ≤0.6; and strong if *r* > 0.6 [19].

The authors assessed the change in global and domain scores of FACIT-F and CS-31 between the three time points (T0, T1, and T2). Differences were evaluated using the One-way ANOVA test with repeated measures and Sidak post-hoc, or the non-parametric Friedman with multiple comparison tests.

The known-group validation analysis was performed to assess if the instrument would be able to discriminate between subgroups of survivors, using one-way ANOVA with Sidak post-hoc. The CS-31 global scores were compared with non-cases, doubtful cases, and cases of anxiety and depression by HADS. The authors hypothesized that those survivors with higher scores for both anxiety and depression would have a worse HRQL. In BC patients, the HRQL is closely related to these psychological disorders [20].

All statistical analyses were performed using IBM SPSS Statistics (Armonk, NY, USA), software package (SPSS Statistics for Windows, version 21.0), considering statistically significant p-values of less than 0.05.

## Results

This prospective study included 89 postmenopausal BC survivors undergoing endocrine therapy. The median (p25‒p75) age was 65 (58.5‒69.5) years, the median time using AI was 29.5 (18.1‒41.8) months, the median diagnosis time was 4 (2‒5) years, and the median climacteric period was 16 (8‒20) years. Most survivors were not married (56.2%, n = 50), but most had a partner (75.3%, n = 67). Regarding adjuvant endocrine therapy, 44.9% (n = 40) of survivors used tamoxifen prior to starting AI. The demographic and clinical characteristics are presented in Table 1.

**Table 1:** Demographic and clinical characteristics of the breast cancer survivors during endocrine therapy.

Characteristics	Overall (n = 89)
Age (years)	65 (58.5‒69.5)
< 60	25 (28.1)
≥ 60	64 (71.9)
Marital Status	
Single/ Divorced/Separated/Widow	50 (56.2)
Married	39 (43.8)
Partner	
No	22 (24.7)
Yes	67 (75.3)
Educational Level	
Below high school	61 (68.5)
High school or higher education	28 (31.5)
Income (minimum wage)	
< 3	53 (59.6)
≥ 3	36 (40.4)
Work activity	
Active	22 (24.7)
Inactive	67 (75.3)
Surgery	
Breast-conserving surgery	51 (57.3)
Mastectomy	38 (42.7)
Prior Radiotherapy	
No	14 (15.7)
Yes	75 (84.3)
Prior Chemotherapy	
No	21 (23.6)
Yes	68 (76.4)
Chemotherapy Regimen	
Adjuvant	53 (77.9)
Neoadjuvant	15 (22.1)
Prior Tamoxifen	
No	49 (55.1)
Yes	40 (44.9)
Tumoral Subtype	
Ductal	86 (96.6)
Lobular	3 (3.4)
Clinical Stage	
I	26 (29.2)
II	48 (53.9)
III	13 (14.6)
NR	2 (2.2)
Tumor Grade	
G1	14 (15.7)
G2	66 (74.2)
G3	5 (5.6)
NR	4 (4.5)
Molecular Subtype	
ER+ and/or PR+, HER2- and Ki-67 < 14%	17 (19.1)
ER+ and/or PR+, HER2- and Ki-67 ≥ 14%	37 (41.6)
ER+ and/or PR+, HER2+	29 (32.6)
NR	6 (6.7)
Months since start of AI	29.5 (18.1‒41.8)
Years since diagnosis	4 (2‒5)
Years since last menstrual period	16 (8‒20)

Continuous variables are shown as median (p25-p75), and categorical variables are shown as absolute numbers and percentage frequency (in parentheses); Time point: T0, Initial follow-up period; Prior, before starting AI use; AI, Aromatase Inhibitor; ER, Estrogen Receptor; PR, Progesterone Receptor; HER2, Human Epidermal growth factor type 2 Receptor; Ki-67, Ki-67 antigen; -, negative; +, positive; NR, Not Reported; G1, Well-differentiated tumor (low grade); G2, Moderately differentiated tumor (intermediate grade); G3, Poorly differentiated tumor (high grade). The Brazilian minimum wage was R$ 880.00.

### Internal consistency

The CS-31 presented good global internal consistency (Cronbach's alpha = 0.89), without significant change if a single item was deleted (Table 2).

**Table 2:** Cronbach's α of the 31-item Cervantes Scale (CS-31).

Domain	Cronbach's α (95% CI)	Item	Descriptors	Mean (SD)	Cronbach's α if item deleted
Menopause and Health (n = 89)	0.81 (0.75–0.86)	1	During the day, I feel my head hurts more and more	1.38 (1.54)	0.799
		11	Even when I sleep, I cannot rest	2.72 (1.91)	0.786
		14	I believe I'm retaining fluid, because I'm swollen	1.98 (1.90)	0.800
		16	I have aching in muscles and/or joints	3.38 (1.39)	0.798
		18	I am afraid of performing physical efforts because my urine leaks	0.97 (1.66)	0.805
		23	I feel my heart beating quickly and out of control	1.80 (1.77)	0.795
		25	My health causes me problems with housework	2.31 (1.95)	0.787
		27	I have vaginal discomfort and dryness	1.71 (1.95)	0.806
		29	I have hot flushes	2.42 (2.01)	0.789
		3	Suddenly, I feel very warm	2.84 (1.89)	0.798
		31	I have noticed skin dryness	3.37 (1.89)	0.813
		5	I cannot get sufficient sleep	3.06 (1.78)	0.797
		7	I feel tingling in my hands and feet	2.19 (1.90)	0.789
		9	Suddenly, I start sweating without effort	2.72 (2.02)	0.797
		-20	I'm as healthy as anyone my age	1.53 (1.52)	0.800
Psychological (n = 89)	0.85 (0.80–0.90)	2	I feel anxious or nervous	2.34 (1.91)	0.831
		6	Everything bothers me, including the things that used to amuse me	1.76 (1.77)	0.839
		10	I lost the ability to relax	2.24 (1.90)	0.836
		12	I feel like the world is spinning around me	1.91 (1.92)	0.852
		17	I believe people are better without me	0.70 (1.46)	0.847
		19	I feel tired since waking up	2.07 (1.94)	0.834
		21	I have the perception of being useless	1.33 (1.76)	0.825
		24	Sometimes I think I wouldn't care if I was dead	1.06 (1.73)	0.847
		28	I feel empty	2.06 (1.99)	0.824
Sexuality (n = 89)	0.84 (0.76–0.88)	4	My interest in sex is the same as always	1.07 (1.65)	0.768
		30	In my life, sex is…	1.58 (1.95)	0.785
		22	I'm having sexual intercourse as often as before	0.69 (1.37)	0.776
		15	I am satisfied with my sexual life	1.71 (2.10)	0.812
Couple Relations (n = 67)	0.75 (0.63–0.84)	8	I consider myself happy in my relationship	2.97 (1.92)	0.619
		26	In my relationship, I feel that I am treated as an equal	3.01 (2.02)	0.592
		13	My role as wife or partner is…	3.49 (1.86)	0.782
**Global Score**^a^	0.89 (0.84–0.92)				

Time point: T0, Initial follow-up period; SD, Standard Deviation; CI, Confidence Interval.

a No single item significantly modified the internal consistency of the CS-31 global and domains when deleted.

### Construct validity

The authors established five a priori hypotheses of strong and moderate convergent analyses between the CS-31 and FACIT-F (global score and domains). Correlations were performed and there was confirmation for all hypotheses tested (Table 3).

**Table 3:** A priori hypotheses and results for construct validity using correlation between the CS-31 and FACIT-F.

Hypothesis	Comparison		***r***	**p**
	Cervantes Scale	FACT-F		
Strong convergent validity expected between similar constructs. Expected correlation *r* > 0.6	Psychological (CS-31)	Emotional Well-Being (EWB)	-0.766	<0.001^a^
Moderate convergent validity expected between items (global scores, constructs indirectly related). Expected correlation *r* > 0.4	Sexuality (CS-31)	Social/Family Well-Being (SWB)	-0.453	<0.001^a^
	Couple Relations (CS-31)	Social/Family Well-Being (SWB)	-0.436	<0.001^a^
	Global Score (CS-31)	FACIT-F Total Score	-0.837	<0.001^b^
	Global Score (CS-31)	FACT-G Total Score	-0.842	<0.001^b^

Time point: T0, Initial follow-up period; CS-31, 31-item Cervantes Scale; FACIT-F Total Score, EWB + PWB + FWB+ SWB + FS; FACT-G Total Score: EWB + PWB + FWB+ SWB. Correlation is significant at the 0.01 level (2-tailed).

a Spearman correlation

b Pearson correlation. Correlations that were consistent with hypotheses are in bold.

### Responsiveness analyses

In Table 4, the authors identified that there was a significant improvement in HRQL by the CS-31 Global score (p = 0.001) as well as by the FACIT-F Total Score (p = 0.044), throughout the study. All the significances indicate worse global and domain scores in T0 compared to T1 and T2, either by CS-31 or FACIT-F, indicating that survivors started endocrine therapy with a worse HRQL. The CS-31 Global score, the Menopause and Health score (p = 0.004) and the Psychological score (p = 0.002) were higher in T0 compared to T1 and T2, and did not differ between T1 and T2. The Emotional Well-Being score (p = 0.029), Physical Well-Being score (p = 0.016) and FACIT-F TOI (p = 0.017) differed statistically between T0 and T2, with higher scores at baseline. The Fatigue Subscale had higher scores at T0 when compared to T1 (p = 0.012). The domains Sexuality and Couple Relations, as well as FACT-G, Functional, and Social/Family Well-Being (FACIT-F) did not differ statistically.

**Table 4:** Variation in the domains and global scores of the CS-31 and FACIT-F over time.

Dependent variables	n	Score range	Mean ± SD or Median (p25-p75)			p-value
			T0	T1	T2	
CS-31						
Menopause and Health	21	0‒75	36 (25.5‒49.5)^a^	28 (20.5‒41.5)^b^	26 (16.5‒42)^b^	0.004
Psychological	21	0‒45	20 (8.5‒27.5)^a^	9 (4.5‒16.5)^b^	14 (4‒22.8)^b^	0.002
Sexuality	21	0‒20	14 (4.5‒20)	15 (8‒18)	14 (7‒20)	0.831
Couple Relations	21	0‒15	3 (0.5‒6.5)	4 (0.5‒7.5)	6 (0.5‒9.5)	0.461
Global Score	21	0‒155	88 (45.5‒94.5)^a^	64 (40‒76)^b^	68 (40‒87)^b^	0.001
FACIT-F						
Emotional Well-Being (EWB)	38	0‒24	18 (14‒21)^a^	18 (15.8‒21)^a,b^	18.5 (16.8‒22)^b^	0.029
Physical Well-Being (PWB)	38	0‒28	19 (16‒23)^a^	21 (16‒26)^a,b^	21.5 (18‒24)^b^	0.016
Functional Well-Being (FWB)	38	0‒28	18.6 ± 5.2	18.5 ± 4.8	18.2 ± 4.6	0.799
Social/Family Well-Being (SWB)	38	0‒28	18.4 ± 5.2	18.4 ± 5.6	17.9 ± 5.1	0.702
Fatigue Subscale (FS)	38	0‒52	35.5 (27‒44.3)^a^	39.5 (32.8‒46.3)^b^	41.5 (35‒45)^a,b^	0.012
FACIT-F Trial Outcome Index (TOI)	38	0-108	73.4 ± 18.1^a^	77.4 ± 17.0^a,b^	78.9 ± 13.6^b^	0.017
FACIT-F Total Score	38	0-160	109.5 ± 24.8	114.0 ± 22.9	115.5 ± 19.3	0.044
FACT-G Total Score	38	0-108	73.7 ± 15.5	75.5 ± 15.1	76.0 ± 13.0	0.364

Time point: T0, Initial follow-up period; T1, Intermediate period, corresponding to 12-months after T0; and T2, Final follow-up period, corresponding to 24 months after T0; CS-31, 31-item Cervantes Scale; FACIT-F, Functional Assessment of Chronic Illness Therapy – Fatigue; FACIT-F Trial Outcome Index (TOI): Physical Well-Being + Functional Well-Being + Fatigue Subscale; FACIT-F Total Score: Emotional Well-Being + Physical Well-Being + Functional Well-Being + Social/Family Well-Being + Fatigue Subscale; FACT-G Total Score: Emotional Well-Being + Physical Well-Being + Functional Well-Being + Social/Family Well-Being. SD, Standard Deviation. One-way ANOVA test with repeated measures and Sidak post-hoc, or the non-parametric Friedman with multiple comparison tests. Bold value is statistically significant at p < 0.05.

### Known-group validity

As previously hypothesized, those survivors with higher scores for both anxiety and depression by HADS presented worse HRQL by CS-31 when compared to subgroup non-cases (Table 5).

**Table 5:** Known-group validation analyses.

Cervantes Scale	Mean ± SD					
	Anxiety (HADS-A)			Depression (HADS-D)		
	Non-cases	Doubtful cases	Cases	Non-cases	Doubtful cases	Cases
Global Score CS-31	53.4 ± 20.9^a^	80.1 ± 20.3^b^	94.7 ± 15.7^b^	56.08 ± 23.4^a^	85.1 ± 16.4^b^	90.6 ± 17.6^b^
N	33	17	18	37	10	21

Time point: T0, Initial follow-up period; CS-31, 31-item Cervantes Scale; SD, Standard Deviation; HADS-A, Hospital Anxiety and Depression Scale, subscale anxiety; HADS-D, Hospital Anxiety and Depression Scale, subscale depression. One-way ANOVA with Sidak post-hoc. All p-values are < 0.001.

## Discussion

The psychometric properties revealed that CS-31 is a valid instrument for assessing HRQL in BC survivors during adjuvant endocrine therapy. The CS presented adequate internal consistency, satisfactory construct validity, and known-group validity, with statistical significance between anxiety and depression and worse HRQL. Furthermore, the authors identified a prospective improvement in HRQL of the baseline for the other time points.

The authors identified higher scores for anxiety and depression in survivors with worse HRQL and improvement in HRQL over the study. Recently, Martino and collaborators [21] identified that after 6-months of treatment with AI, BC patients presented a significantly higher perceived HRQL for both physical and mental components, added to a significant reduction in symptoms of anxiety and depression, possibly due to the decline of the physical and psychological effects of recent diagnosis and previous treatments [21].

Regarding the Sexuality and Couple Relations domains of CS-31, as well as the Social/Family Well-Being of FACIT-F, the latter which also presents items related to sexual life and couple relations, have not changed over time. Often, the adverse effects of treatment, as well as induced menopause, cause sexual dysfunction among BC survivors, with a relevant impact on sexual function [22]. The disturbances in sexual life are among the factors that might deteriorate the quality of life in BC survivors [23]. The adjuvant endocrine therapy, especially AI, can cause vaginal atrophy [24], dryness and dyspareunia [4], and some urogenital effects, and may be lifelong if untreated [25]. Possibly, the treatment has a more lasting impact on sexuality and a longer follow-up would be necessary to investigate changes in these domains. Furthermore, it should be noted that sexuality is considered a biopsychosocial concept, and therefore it is believed to be associated with biological and psychosocial factors [26]. The main recommendation for the management of sexual health in BC survivors is that a multidisciplinary team needs to include sexuality as an integral part of treatment, contributing to an improvement of HRQL [22].

Even though the CS-31 was not designed for this population, these women have predominantly adverse effects like those of other postmenopausal women, although intensified by the AI use. The authors need to consider that the CS-31’s target population is women aged 45 to 64 years[9] and the present sample includes women aged 47 to 79 years. For this purpose, the authors divided survivors into two age groups (47 to 64 and 65 to 79 years) and observed that age had no effect on the CS-31 scores by performing the Generalized Linear Model (GLzM) analysis (data not shown). In addition, the CS-31 is a self-reported questionnaire, however, in this study, all participants replied by interview, which may have inhibited responses to items in the Sexuality and Couple Relations domains. Even so, the standardization for this type of application was a methodological care considering that in the present sample there were illiterate survivors.

As pointed out by others [13], the authors identified that most of the invalid questionnaires were filled out by women who were not married or without a partner, referring to a sexually inactive life. This seems to be a limitation of the CS-31, and adaptations to this instrument are necessary to contemplate all climacteric women, irrespective of their marital status and sexual activity.

Adherence to adjuvant endocrine therapy is suboptimal in BC patients. It is negatively associated with the treatment for adverse events[8] and associated with increased early tumor recurrence and mortality rates [27]. Potentially, clinical interventions to manage these adverse effects may improve HRQL and BC outcomes [28]. CS-31 is multidimensional allowing in-depth analysis of adverse effects and general HRQL. Knowing details that permeate the HRQL of these survivors, certainly, can be useful in clinical management. The strength of the current study includes the use of the CS-31 to assess HRQL at three-time points, with a 2-year follow-up.

The authors must recognize that the small sample size represents a limitation of the present study. Conducting future research with a larger sample size would enable additional analyses, such as factor analysis for construct validity and responsiveness analysis using an anchor-based strategy, thereby enhancing the statistical power of the study. It is important to note that test-retest analysis, a valuable psychometric property, could be incorporated in subsequent studies to further validate the present findings. Another limitation lies in the absence of specification regarding the treatment phase during which each patient was recruited. A future study that examines the impact of this variable on Health-Related Quality of Life (HRQoL) is needed.

As mentioned above, other studies are necessary to confirm whether the implementation of CS-31 in oncology medical routine is able to contribute to improvements in HRQL and in the prognosis of these survivors. Even so, the authors suggest that CS-31 is used in outpatient service to investigate HRQL and in early screening of adverse effects in BC patients in AI use. The authors believe that the use of CS-31 can optimize attendance time and health outcomes, since health professionals could focus on individual adverse effects and monitor, through graphic summaries, the evolution of these effects after specific interventions.

## Conclusions

The authors identified that the CS-31 seems to be an appropriate instrument for use in oncology medical routine with BC survivors during adjuvant endocrine therapy and may help to monitor adverse effects and HRQL, although larger studies are needed to confirm these results.

## Authors’ contributions

Isis Danyelle Dias Custódio, Carlos Eduardo Paiva e Yara Cristina de Paiva Maia made substantial contributions to the conception and design of the study, data acquisition, analysis and interpretation, manuscript drafting and critical review for important intellectual content. Fernanda Silva Mazzutti Nunes, Mariana Tavares Lima, Kamila Pires de Carvalho, Andressa Miranda Machado and Paula Philbert Lajolo made substantial contributions to the data acquisition, analysis and interpretation, manuscript drafting and critical review for important intellectual content. All the authors have approved the final version of the manuscript.

## Funding

This study received financial support from Conselho Nacional de Desenvolvimento Científico e Tecnológico, Brasil (CNPq Grant number: 409482/2021-8); Federal University of Uberlândia, Programa de Pós-graduação em Ciências da Saúde; Fundação de Amparo à Pesquisa do Estado de Minas Gerais (FAPEMIG) Grant number: APQ-01339-21; Fundação de Amparo à Pesquisa do Estado de Minas Gerais (FAPEMIG) Grant number: APQ-01961-23 and CAPES. Yara Cristina de Paiva Maia is supported by a grant from Fundação de Amparo à Pesquisa do Estado de Minas Gerais - FAPEMIG [Rede Mineira de Pesquisa Translacional em Imunobiológicos e Biofármacos no Câncer (REMITRIBIC, RED-00031-21)]. The funders had no role in study design, data collection and analysis, decision to publish, or preparation of the manuscript.

## Ethics approval and consent to participate

This study was approved by the Human Research Ethics Committee of Federal University of Uberlandia (n 1.331.949/15, addendum n 2.905.835/18) and conducted based on the norms of the Declaration of Helsinki. All participants signed a free and informed consent.

## Declaration of competing interest

The authors declare no conflicts of interest.
